# Therapeutic Potential of Peptides Derived from Animal Venoms: Current Views and Emerging Drugs for Diabetes

**DOI:** 10.1177/11795514211006071

**Published:** 2021-03-27

**Authors:** Aimee Coulter-Parkhill, Stephen McClean, Victor A Gault, Nigel Irwin

**Affiliations:** Diabetes Research Group, Ulster University, Coleraine, UK

**Keywords:** Clinical trials, diabetes, exendin-4, venom therapeutics

## Abstract

The therapeutic potential of venom-derived drugs is evident today. Currently, several significant drugs are FDA approved for human use that descend directly from animal venom products, with others having undergone, or progressing through, clinical trials. In addition, there is growing awareness of the important cosmeceutical application of venom-derived products. The success of venom-derived compounds is linked to their increased bioactivity, specificity and stability when compared to synthetically engineered compounds. This review highlights advancements in venom-derived compounds for the treatment of diabetes and related disorders. Exendin-4, originating from the saliva of Gila monster lizard, represents proof-of-concept for this drug discovery pathway in diabetes. More recent evidence emphasises the potential of venom-derived compounds from bees, cone snails, sea anemones, scorpions, snakes and spiders to effectively manage glycaemic control. Such compounds could represent exciting exploitable scaffolds for future drug discovery in diabetes, as well as providing tools to allow for a better understanding of cell signalling pathways linked to insulin secretion and metabolism.

## Introduction

Crude animal venom contains a diverse mixture of bioactive compounds that target a variety of receptors to support survival of venomous animals.^1^ However, venoms and their metabolites are now being recognised as potential exploitable tools in medicine.^2,3^ As such, the term ‘venomics’ was first used by Juárez et al^4^ to describe characterisation of the complete protein profile of snake venom. Following on from this, more recent advances in analytical techniques including incorporation of genomics, mass spectrometry and proteomics have assisted scientists to more easily explore venom profiles.^5^ Together with modern-day ability to rapidly screen venom compounds using high-throughput assays, this represents a step-change in realising the full therapeutic potential of animal venoms.^5^ This review summarises current clinically approved venom-based drugs, and briefly considers venom-derived drugs in clinical trials as well as use of venom products as cosmeceuticals before finally highlighting the therapeutic promise of such compounds for obesity-diabetes.

## Clinically Approved Venom-Derived Drugs

### Captopril and enalapril

Ferreira et al^6^ purified and characterised bradykinin-potentiating factors (BPFs) from the Brazilian viper snake venom, which the Squibb Institute of Medical Research was then able to utilise teprotide, which acted as an ACE inhibitor.^7^ After studying structure/function aspects of teprotide, the orally active compound named SQ 14 225 (D-2-methyl-3-mercaptopropanoly-L-proline), now better known as captopril was generated (Table 1), and was the first ACE inhibitor that effectively lowers blood-pressure in humans.^7-9^ Following captopril, enalapril was synthesised by the substitution of the mercapto group present on the captopril structure, to an alkyl group.^10^ Despite limited oral bioavailability, the prodrug displays good potency and was clinically approved in 1985 as Vasotec, for the treatment of hypertension and congestive heart failure.^11-13^

**Table 1.** Origin, treatment indication and current methods for production of currently clinically approved venom-derived drugs.

Drug	Species	Treatment indication	Production
Captopril and Enalapril (Capoten, Vasotec)	Brazilian viper, *Bothrops jararaca*	Hypertension and related cardiovascular disorders	Synthetic
Ziconotide (Prialt)	Cone snail, *Conus magus*	Chronic pain	Synthetic
Eptifibatide and Tirofiban (Integrilin, Aggrastat)	Saw-scaled viper, *Echis carinatus*	Thrombotic cerebrovascular or cardiovascular disease	Synthetic
	Pygmy rattle snake, *Sistrurus miliarius*		
Lepirudin and Bivalirudin (Refludan, Angiomax)	Blood-sucking leech, *Hirudo medicinalis*	Stroke, deep vein thrombosis and pulmonary embolism	Synthetic
Batroxobin (Defibrase)	Brazilian lancehead snake, *Bothrops moojeni*	Stroke and ischemic attack and sudden deafness	Purified from venom
Bee venom therapy (Apitox)	Bee, *Apis mellifera*	Osteoarthritis	Whole venom
Exenatide (Byetta, Bydureon)	Gila Monster lizard, *Heloderma suspectum*	Type 2 diabetes	Synthetic
Cobratoxin (Cobratid)	Chinese cobra, *Naja naja atra*	Pain	Purified from venom

### Ziconotide

Ziconotide is an intrathecal analgesic drug that was clinically approved in 2004 for the treatment of chronic pain^14^ (Table 1). Marketed as Prialt, this 25 amino acid peptide is a synthetic version of ω-conotoxin MVIIA (ω-MVIIA), extracted from the venomous cone snail *Conus magus*.^14-16^ It has been demonstrated that ω-conotoxin blocks *N*-type voltage gated calcium channels and prevents the release of pro-nociceptive neurochemicals including glutamate, calcitonin gene-related peptide and substance *P*.^17-19^ Prialt is also used to treat chronic pain in those with intolerances to morphine or other systemic analgesics.^20^

### Eptifibatide and tirofiban

Tirofiban was the first approved venom-derived antiplatelet drug based on the structure of echistatin, a peptide extracted from venom of the saw-scaled viper, *Echis carinatus*^21^ (Table 1). Echistatin, isolated in 1988, is a glycoprotein (GP) IIb/IIIa receptor antagonist leading to effective inhibition of fibrinogen-induced platelet aggregation.^21-25^ Furthermore, Scarborough et al^26^ screened 62 snake venoms, leading to the discovery of barbourin from the pygmy rattle snake, *Sistrurus miliarius*, that specifically inhibited GP IIb/IIIa receptors. These findings ultimately led to the synthesis of eptifibatide (Table 1), a highly effective antiplatelet drug.^27,28^

### Lepirudin and bivalirudin

Lepirudin and bivalirudin (Table 1) are specific thrombin inhibitors, with structures based on hirudin, a peptide with strong anticoagulant properties found in blood-sucking leeches, Hirudo medicinalis.^29,30^ Lepirudin was clinically approved in 1998, and bivalirudin followed in 2000.^31^ In 2012, lepirudin was discontinued, although this was not due to inefficacy or adverse effects, but rather manufacturing issues.^32^ Bivalirudin, on the other hand is still routinely prescribed to treat unstable angina and percutaneous coronary intervention.^33-35^

### Batroxobin

Batroxobin (Table 1), isolated from the venom of the *Bothrops moojeni* venomous snake is a thrombin-like serine protease which is a defibrinogenating agent with anti-inflammatory effects.^36,37^ Batroxobin is a 231 amino acid protein that helps to form non-cross-linked fibrin clots and subsequently release fibrinopeptides *A*.^38,39^ Purified from *B. Moojeni* venom, it is marketed as Defibrase and currently available for use in China for the treatment of stroke and ischemic attack.^40,41^

### Apitox

Bee venom extracted from *Apis mellifera* and marketed as Apitox has been clinically approved in South Korea for the treatment of osteoarthritis^42,43^ (Table 1). This approval came after a phase III clinical trial with 363 patients with knee osteoarthritis.^44^ Furthermore, Apimeds are sponsoring a clinical trial for use of apitox in the treatment of Multiple Sclerosis (MS), as the drug is believed to be effective in reducing pain and swelling associated with the disease but there are no clinical data currently available in this regard.^45^

### Cobratid

A short-chain α-neurotoxin isolated from cobra venom *Naja naja atra*, is known to act as an antagonist of nicotinic acetylcholine receptors (nAChRs) and bring about analgesic effects.^46^ As such, in a rodent model of pain, cobratid exerted dose-dependent analgesic effects that were independent of muscarinic ACh and opioid receptor modulation.^47^ Furthermore, cobratid, now promoted as a tablet named Keluoqo that also contains tramadol hydrochloride and ibuprofen, has undergone clinical trials for the treatment of moderate to severe pain associated with cancer.^48^ Whilst results of this study were promising, issues around development of tolerance still need to be addressed. However, it should be noted that studies are ongoing in a bid to help minimise adverse effects associated with use of this drug.^49-51^

### Exenatide

**Figure 1.**
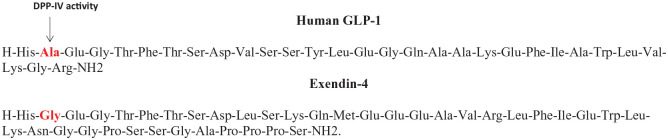
The amino acid sequence of exenatide in comparison to native GLP-1. The key amino acid substitution at position 2 that confers full resistance to DPP-4 in exenatide is highlighted.

**Figure 2.**
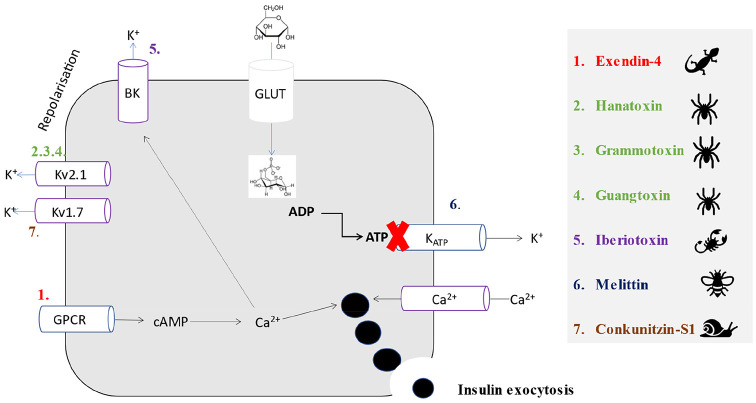
A schematic showing proposed insulin secretory pathways for the venom-derived peptides hanatoxin grammotoxin, guangtoxin, exendin-4, conkunitzin-S1, iberiotoxin and melittin within pancreatic beta cells. A simplified pathway for the secretion of insulin via the primary beta cell stimulus, namely glucose, is depicted for reference via conversion to glucose-6-phospahate, with subsequent generation of ATP and closure of K_ATP_ channels. Colour coded numbers correspond to proposed beta-cell membrane target of each venom-derived peptide, with the specific K^+^ channel that melittin binds to still not known. Indeed, the mechanisms of all peptides, barring exendin-4, still needs to be fully characterised. Abbreviations: ADP, adenosine diphosphate; ATP, adenosine triphosphate; BK, calcium-activated (big) potassium channel; cAMP; cyclic adenosine monophosphate; GLUT, glucose transporter 2; GPCR, G-protein coupled receptor; Kv, voltage-gated potassium channel.

Oral glucose administration results in a greater insulinotropic response compared to similar intravenous glucose delivery in humans.^52^ This phenomenon, known as the ‘incretin effect’, is related to the secretion of the gut-derived hormones glucagon-like peptide 1 (GLP-1) and glucose-dependent insulinotropic polypeptide (GIP).^53^ Both hormones act on specific pancreatic beta cell G-protein-coupled receptors (GPCRs), that stimulate adenylyl-cyclase activity, cAMP accumulation and ultimately insulin secretion.^54^ In type 2 diabetes, secretion of GLP-1 from the GI tract is blunted following nutrient intake highlighting an ideal drug target for the disease.^55^ However, native GLP-1 has a short biological half-life due to degradation by the ubiquitous enzyme dipeptidyl peptidase-4 (DPP-4) upon secretion into the bloodstream, limiting therapeutic application of this peptide. DPP-4 has a wide substrate specificity and acts on various regulatory peptides, chiefly cleaving small peptides with proline or alanine as the penultimate *N*-terminal residue.^56^ As such, although DPP-4 inhibitor drugs are now clinically approved for the treatment of type 2 diabetes with an excellent adverse effect profile, lack of substrate specificity was an initial concern.^56^ In this respect, the first clinically approved GLP-1 peptide mimetic with inherent stability against DPP-4 was derived from the saliva of the venomous Gila monster lizard, *Heloderma suspectum*.^57,58^ The peptide, first isolated by Eng et al^57^ was named exendin-4 and shown to have 53% homology to human GLP-1. Importantly, exendin-4 possesses an amino acid substitution at the penultimate *N*-terminal residue that masks the DPP-4 binding site and dramatically extends the duration of biological action of the peptide^58^ (Figure 1; Table 1). Exendin-4 was initially marketed as Byetta (Figure 2), and then later as an extended release preparation known as Bydureon, which were approved for clinical use in 2005 and 2017, respectively, for the treatment of type 2 diabetes.

## Venom and Cosmeceuticals

Cosmeceuticals represent an attractive avenue for commercial use of venom-derived products. This is typified by the global success of Botulinum toxin (Botox) based therapies for medical and particularly cosmetic purposes, where Botox represents a type A toxin isolated from *Clostridium botulinum* bacteria.^59^ The toxin acts on neuromuscular junctions to prevent acetylcholine (ACh) release from presynaptic neurons, subsequently causing mild muscle paralysis.^60^ Indeed, since first use of Botox for a cosmeceutical procedure to decrease the appearance of wrinkles in 1989, the drug now generates around $3 billion per annum.^61,62^ In a separate venture, snake venom has also entered the cosmeceutical industry for use in facial serums to reduce wrinkles. An example of this being the use of synthetic tripeptide (Tripeptide [SYN®-AKE]) from *Tropidolaemus wagleri* snake venom. Tripeptide represents a synthetic version of waglerin 1, which also blocks the action of ACh to subsequently inhibit muscle contraction.^63^ Additionally, venom purified from the *Apis mellifera L.* honeybee is also currently being exploited in facial serums as it is believed to reduce total wrinkle size in individuals with UV damage.^64^ Most recently, argiotoxine-636 (ArgTX-636) isolated from the *Argiope lobata* spider venom has been patented for its use in skin and teeth whitening.^65^ Although the mechanism of action of ArgTX-636 remains to be fully elucidated, inhibitory effects on melanogenesis is believed to be key in these cosmeceutical applications.^66^ In terms of approval processes, it is important to note the differences in regulatory authorisation for a drug and a cosmeceutical. As such, a cosmeceutical is considered to exert a pharmaceutical therapeutic benefit but not necessarily linked to an established biological benefit.^67^ Therefore, unlike drugs, the FDA does not necessarily review or approve cosmeceuticals prior to sale, representing a much simpler approval process than with drugs. However, there is still some controversy around the exact boundaries of what constitutes a cosmeceutical and a drug,^68^ that requires further detailed clarification.

## Venom-Derived Drugs in Clinical Trials

### Early clinical trials

There has been much effort made to utilise natriuretic peptides, namely atrial natriuretic peptide (ANP), ventricular natriuretic peptide (BNP) and C-type natriuretic peptide (CNP) for the treatment of heart failure.^69-71^ The first characterised reptilian natriuretic peptide, dendroaspis natriuretic peptide (DNP), was extracted from the Eastern green mamba snake *Dendroaspis angusticeps*.^72^ DNP, a 38 amino acid peptide that has structural similarities to human natriuretic peptides, is a potent activator of cardiomyocyte guanylate cyclase *A*, which aids in cardiac unloading and therefore may have benefits in heart failure.^73,74^ Cenderitide, a derivative of DNP, was synthesised and developed by the Mayo Clinic through fusion of the 22 amino acid peptide CNP with the 15 amino acid *C*-terminal of DNP.^74^ A clinical trial was conducted in 2015 by Capricor, to assess efficacy and safety of subcutaneous infusion of cenderitide in subjects with stable, chronic heart failure.^75^ Although the drug was well tolerated, further clinical assessment of cenderitide by Capricor has not been carried out. However, a phase I clinical trial by Mayo Clinic with cenderitide in 30 participants with myocardial infarction was completed in 2019.^76^

Another approach for utilising snake venom involved the extraction of the enzyme metalloproteinase fibrolase from the southern copperhead snake *Agkistrodon contortrix contortrix*.^77,78^ The protein consisting of 203 amino acids, underwent DNA recombination to create alfimeprase, a compound which directly degrades fibrin to result in thrombolysis.^79-81^ Alfimeprase successfully underwent a phase I clinical trial in patients with acute peripheral arterial occlusion (PAO) and was well tolerated with no reports of bleeding or systemic thrombolysis.^82^ Although alfimeprase progressed to phase II and III trials, these were terminated as there was ultimately no significant improvement when compared to placebo.^82,83^

The discovery and success of ziconotide from *Conus magus* has brought much attention to the potential of marine venom-derived compounds. Therefore, it is not surprising there has been the extraction and characterisation of such peptides, progressing into clinical trials. Firstly, Vc1.1 a 16 residue α-conotoxin (ACV1) from *Conus victoriae* is a potent antagonist of nAChRs, where modulation of nAChR’s is associated with clear analgesic actions.^84,85^ Metabolic Pharmaceuticals conducted a phase I human clinical trial with ACV1 in healthy males, where it was well tolerated, and no adverse effects reported.^86^ However, lack of efficacy in phase IIa trials for diabetic peripheral neuropathic pain led to further clinical research with ACV1 being discontinued, but α-conotoxins are still being explored as future drugs for the treatment of neuropathic pain.^87-89^

In another attempt to exploit a conotoxin with analgesic properties, the χ-conotoxin χ-MrIA, was extracted from the *Conus marmoreus*.^90^ The 13-residue peptide was found to non-competitively inhibit the norepinephrine transporter (NET) in humans,^91^ whereby inhibiting the reuptake of norepinephrine has demonstrated analgesic properties.^91^ Thus, Xen2174, a synthetic version of χ-MrIA with the addition of a pyroglutamyl residue to the *N* terminus, demonstrated analgesic effects in a rat model of neuropathic pain.^92^ Whilst Xen2174 displayed promising results in a phase II clinical trial for cancer patients with chronic pain, it ultimately failed to pass into phase IIb due to dose-limiting toxicity.^2^ Lastly, contulakin-G is a 16 amino acid peptide extracted from the *Conus geographus and was shown to inhibit neurotensin receptors (NTRs).^93,94^ Neurotensin is a peptide found in the CNS with an influence on the internal* analgesic system. 95*In this regard*, contulakin-G, later named CGX-1160, received orphan drug status in 2005, after the success of a phase Ib clinical trial for the treatment of neuropathic pain associated with spinal cord injury, but has since been discontinued.^96^

Another venomous animal with perceived therapeutic value is the vampire bat *Desmodus rotundus*, specifically through isolation of a 441 amino acid fibrin-dependent plasminogen activator from its venom.^97,98^ Later named desmoteplase, this thrombolytic agent effectively breaks down blood clots by converting plasminogen to plasmin.^97^ Desmoteplase was investigated as a potential therapeutic agent for ischemic stroke,^99^ however, despite promising initial observations, desmoteplase was subsequently terminated following lack of prominent efficacy in a phase III clinical trial.^100-102^

### Current clinical trials

#### Cancer

Chlorotoxin is a 36 amino acid peptide extracted from the venom of the Deathstalker scorpion, *Leiurus quinquestratius*,^103^ which has shown specificity towards various cancerous cell lines including glioma, melanoma and carcinoma.^104^ It is theorised that chlorotoxin can specifically target chloride ion channels on glioma tumour cells to prevent malignancy and decrease tissue invasion potency.^105,106^ As of January 2020, the City of Hope Medical Centre in California began recruiting participants for phase I testing of the safety and efficacy of chlorotoxin for recurrent glioblastoma cancer.^107^ The trial will involve the delivery of chimeric antigen receptor (CAR-T lymphocytes), with chlorotoxin as the tumour targeting domain. CAR-T lymphocytes are synthetic *T* cell receptors that can recognise tumours and activate an immune response specific to the cancerous cells.^108^ An additional use of chlorotoxin is with tozuleristide (Table 2), a tumour targeting agent that comprises chlorotoxin and the fluorescent dye, indocyanine green that provides intraoperative visualisation of tumours.^109,110^ Tozuleristide successfully underwent a phase I clinical trial in 17 patients diagnosed with glioma, treatable by surgical excision. The trial revealed no adverse effects and good tolerability of tozuleristide, as well as being highly effective in glioma imaging.^111^ More recently, Blaze Bioscience Inc. is recruiting paediatric patients with CNS tumours undergoing surgery for use of tozuleristide in phase II and III trials.^112^

**Table 2.** Details of venom-derive drugs undergoing clinical trials including name, origin, treatment indication and clinical trial phase progression.

Drug	Species	Treatment indication	Clinical trial phase
Chlorotoxin (CTx)	Deathstalker scorpion, *Leiurus quinquestratius*	Recurrent glioblastoma cancer	Phase 1 [NCT04214392]
Chlorotoxin with tozuleristide (BLZ-100)	Deathstalker scorpion, *Leiurus quinquestratius*	Central nervous system tumours	Phase I and phase II [NCT03579602]
SOR-C13 (Sorcidin)	Northern short-tailed shrew, *Blarina brevicauda*	Advanced refractory solid tumours	Phase Ib [NCT03784677]
ShK-186 (Dalazatide)	Caribbean sea anemone, *Bunodosoma granulifera*	Lupus and erythematous	Phase II
RPI-78M (Receptin)	Chinese cobra, *Naja naja*	Multiple sclerosis and adrenomyeloneuropathy	Phase II and phase III
RPI-MN (Pepteron)	Chinses cobra, *Naja naja*	Amyotrophic lateral sclerosis, herpes simplex keratitis and human immunodeficiency virus	Phase I and phase II

In an alternative approach, cancer cell growth has been linked to increased ambient calcium levels, with the Transient Receptor Potential Vanilloid channels (TRPV6) known to play a key role in regulating normal calcium homeostasis.^113^ Overexpression of TRPV6 within some cancerous cell lines such as breast, pancreatic and ovarian has been detected.^113,114^ Interestingly, a peptide named SOR-C13 (Table 2), modelled on a 253 amino acid protein extracted from the venom of the northern short-tailed shrew, *Blarina brevicauda*, has been shown to prevent calcium uptake via TRPV6.^115^ As such, a clinical trial enrolling patient with solid tumours such as pancreatic and ovarian,^116^ ultimately lead to SOR-C13 being awarded orphan drug status for both ovarian and pancreatic cancer.^116^ More recently, the Anderson Cancer Centre in Texas is currently recruiting patients with advanced refractory solid tumours for treatment with SOR-C13. This is a phase Ib trial that will determine the most effective dose of SOR-C13 in these patients.^117^

#### Autoimmune disease

As well as cancer, there are also ongoing clinical trials with venom-derived compounds in auto-immune disease. In this regard, in 1996 a 35 amino acid peptide called Stichodactyla toxin (ShK) was extracted from a Caribbean Sea anemone *Bunodosoma granulifera* and shown to block Kv1.3 potassium channels^118^ (Table 2). ShK-186, a 37 amino acid analogue of ShK, now known as dalazatide, successfully underwent a Phase I trial consisting of 32 healthy volunteers, displaying only mild adverse effects and good tolerability.^119^ In addition, in a Phase Ib trial for psoriasis in 2014, dalazatide was extremely well tolerated.^120^ This is particularly encouraging given the current lack of effective treatment options for those with autoimmune disease, and the increasing prevalence of the disease worldwide. Furthermore, TEKv Therapeutics (Columbus, OH) are currently preparing Phase II clinical trials for the treatment of autoimmune diseases such as lupus and erythematous with dalazatide.

Additionally, cobratoxin and cobrotoxin from *Naja atra* cobra snake venom has entered clinical trials for the treatment of a range of diseases. Cobratoxin and cobrotoxin have been detoxified to produce chemically modified versions, namely RPI-78M (Receptin) and RPI-MN (Pepteron), respectively (Table 2). The peptides displayed analgesic effects by specifically targeting nAChRs.^121-123^ Additionally, RPI-78M proved safe and effective in a phase I clinical trial for the treatment of MS.^2^ RPI-MN has also proved effective in a preclinical study against the human immunodeficiency virus, as it has shown an ability to inhibit viral replication.^124^

## Emerging Venom-Derived Drugs for Type 2 Diabetes

### Overview of type 2 diabetes

Type 2 diabetes is a complex metabolic disorder characterised by insulin resistance, as well as a decrease in pancreatic beta cell mass and insulin secretion, that ultimately leads to overt hyperglycaemia.^125,126^ If the disease is poorly managed several complications can arise, including cardiovascular and kidney disorders, blindness and neurogenerative disorders.^127,128^ Diabetes is described as one of largest epidemics of the 21st century with the prediction that 642 million people will suffer from diabetes by 2040.^129^ The treatment for obesity-related diabetes is initially education and lifestyle intervention, but such strategies are often ineffective, and patients inevitably progress to pharmacological treatment.^130^ There is an array of drugs available to treat 2 diabetes that can improve insulin secretion (sulfonylurea and meglitinide) or sensitivity (thiazolidines and metformin), enhance the incretin effect (GLP-1 mimetics and DPP-4 inhibitors), increase glucose excretion (sodium glucose transporter 2 inhibitors) or ultimately insulin replacement therapy as the last line of treatment.^131,132^ Although these drugs are effective, they are often associated with adverse effects such as weight gain and hypoglycaemia.^131^ Additionally, drug failure over time is commonplace with type 2 diabetes therapeutics, leading to polypharmacy and ineffective blood glucose control.^132,133^ Therefore, it is essential that new pharmacological agents are developed to provide more effective treatment for diabetes, with less adverse effects that should improve compliance and overall clinical outcomes.

The discovery of exendin-4 from the venom of the Gila monster, as noted above, has brought much attention and acceptability to the use of venom-derived drugs in the field of diabetes therapies. Unlike other antidiabetic agents, exendin-4 has much fewer side effects such as hypoglycaemia and encourages weight loss.^134^ Additionally, it has been demonstrated that exendin-4 increases beta cell proliferation and protects against cardiovascular disorders.^135,136^ To date, products derived from the venoms of bees, cone snails, sea anemones, scorpions, snakes and spiders are being actively investigated as new and effective therapeutic approaches for diabetes,^137^ with exendin-4 recognised as clear proof-of-concept for this exciting drug discovery route.

### Cone snail insulin

Human insulin is composed of 2 peptide chains linked by 2 disulphide bonds, chain A contains 21 amino acids and chain B which contains 30 amino acids.^138^ Con-Ins *G* is an insulin molecule derived from the *Conus geographus* cone snail that lacks the *C*-terminus of the B chain but can activate human insulin receptors.^139,140^ Additionally, unlike human insulin, Cons-Ins G1 has a lower affinity for the primary binding site on the human insulin receptor (hIR), with a preferential affinity for the secondary binding site, suggesting an alternative mechanistic approach to hIR activation.^141^ Furthermore, this insulin peptide contains post-translational modifications in the A and B chain, namely a γ-carboxylated glutamate residue and a hydroxylated proline residue, respectively, which have been hypothesised to increase biological activity.^141^ In this regard, the crystal structure of Con-Ins G1 in comparison to human insulin has been described.^141^ The discovery of Cons-Ins G1 has led to the synthesis of a new recombinant insulin analogue, with an extremely fast onset of action due to its smaller size.^141^ Moreover, the small size of the peptide means chemical synthesis is less complicated, making it a prime candidate for the development of a new insulin therapeutic regimen for humans^141^ (Table 3). Thus, Cons-Ins G1 could represent another important option within the array of clinically approved insulin analogues or could have use in premixed insulin combinations.^137^

**Table 3.** Name, amino acid sequence and origin of venom-derived drugs with reported antidiabetic efficacy.

Species		Toxin name	Sequence	Uniprot	Reference
Tarantula, *Grammostola rosea*	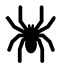	Hanatoxin	ECRYLFGGCKTTSDCCKHLGCKFRDKYCAWDFTFS	P56852	Swartz and MacKinnon^181^
Tarantula, *Grammostola rosea*	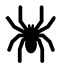	Grammotoxin	DCVRFWGKCSQTSDCCPHLACKSKWPRNICVWDGSV	P60590	Takeuchi et al^186^
Tarantula, *Plesiophrictus guangxiensis*	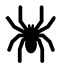	Guangtoxin	DEGECGGFWWKCGSGKPACCPKYVCSPKWGLCNFPMP	P84835	Tilley et al^188^
Cone snail, *Conus geographus*	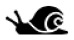	Cons-Ins^a^	A chain: GVV**y**HCCHRPCSNAEFKKYC	A0A0B5AC95	Safavi-Hemami et al^139^
			B chain: TFDT**O**HRCGS**y**ITNSYMDLCYR		
Cone snail, *Conus striatus*	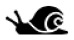	Conkunitzin-S1	KDRPSLCDLPADSGSGTKAEKRIYYNSARKQCLR	P0C1X2	Finol-Urdaneta et al^143^
			FDYTGQGGNENNFRRTYDCQRTCLYT		
Social wasp, *Agelaia pallipes pallipes*	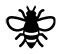	Agelaia MP-1	INWLKLGKAIIDAL	P69436	Baptista-Saidemberg et al^165^
Eastern Indian red scorpion, *Buthus tamulus*	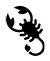	Iberiotoxin	**Pyr**FTDVDCSVSKECWSVCKDLFGVDRGKCMGKKCRCYQ	P24663	Galvez et al^179^

a In Con-Ins, the y indicates a carboxyglutamic acid residue and the O indicates an hydroxyproline residue.

In a separate approach to utilise cone snail venom, there was the isolation of Conkunitzin-S1 (Conk-S1) form the cone snail *Conus striatus*.^142^ This peptide was shown to specifically inhibit Kv1.7 beta cell channels (Figure 2), which resulted in an increase in glucose-stimulated insulin secretion from rat islets. However, unlike current insulinotropic therapeutics such as sulfonylureas and meglitinides, conk-S1 is not associated with hypoglycaemia as glucose-dependent stimulation of insulin secretion has been observed.^143^ The NMR-derived solution structure of recombinant Conk-S1 has been revealed, exhibiting 2 disulphide bonds.^142^ As such, conk-S1 represents a tool to help characterise Kv1.7 channel mechanisms involved in insulin secretion and could also prove to be a valuable novel therapeutic option for type 2 diabetes.^143^

### Caribbean sea anemone

As well as their role in the immune system, Kv1.3 channels have been implicated in the development of insulin resistance and subsequently type 2 diabetes.^144^ This is evident from Kv1.3 gene deletion studies in mice, resulting in enhanced peripheral insulin sensitivity and anti-satiety effects.^145^ Thus, Shk-186 was administered to diet-induced obese mice, resulting in normalisation of blood glucose and insulin, as well body weight reduction.^146^ The anti-obesity mechanism of action has not been fully established. However, it has been suggested that Shk-186 contributes to improved peripheral insulin sensitivity through activation of brown adipose tissue, or via reduction of obesity-induced inflammation of abdominal white adipose tissue.^146^ These results were similar to that observed with Kv1.3 gene deletion, supporting the role of Kv1.3 in insulin resistance.^145^ However, it should also be noted that Kv1.3 channel blockade did not reduce body weight gain in diet-induced obese rats,^147^ and whilst this might be related to subtle differences in dosing regimens and animal models employed, it does necessitate further study on the anti-obesity potential of Kv1.3 inhibition. Although, it has already been demonstrated in clinical trials that ShK-186 has a good safety profile, further promoting potential therapeutic promise for obesity and insulin resistance.

### Snake venom

Poor glycaemic control in diabetes can lead to an increased risk of damage to blood vessels in the eyes known as diabetic retinopathy.^148^ Indeed, type 2 diabetes is now the leading cause of blindness in the UK.^149^ Blindness usually arises due to an angiogenesis cascade leading to neovascularisation from the retinal vessel in a process called choroidal angiogenesis.^150^ In this regard, integrins are receptors that have been implicated in angiogenesis.^150^ Lebecetin is a C-type lectin extracted from the blunt-nose viper snake, *Macrovipera lebetina*, which has been shown to interact with α5β1- and αv-containing integrins.^150^ C-type lectins represent a class of snake proteins which have been implicated in anticoagulant- and platelet-modulating activities. In one study, lebecetin is demonstrated to reduce angiogenesis in a chorioallantoic membrane assay and effectively decrease the extent of choroidal neovascularisation.^150^ Importantly, lebecetin was specific towards the proliferating vascular cells rather than the mature blood vessels suggesting it would be a safe and effective treatment for choroidal angiogenesis.^150^

Sulfonylureas and meglitinides are approved classes of type 2 diabetes drugs that target the ATP-sensitive potassium (K_ATP_) channel on pancreatic beta cells to promote insulin secretion.^151^ However, a major disadvantage of these insulin secretagogues is non-glucose dependent insulin release and subsequent hypoglycaemia.^152^ Thus, constituents of snake venom could yield products that help give a better understanding of these beta cell signalling pathways, leading to drug modifications and a reduction in side effect profiles. As such, the venom from the monocle cobra snake, *Naja kaouthia*, was analysed and fractioned, leading to the isolation of Cardiotoxin-1.^153^ Cardiotoxins have previously demonstrated cytotoxicity and cell damage however, this peptide was demonstrated to be non-toxic to the rodent beta cell-line INS-1.^153^ Cardiotoxin-1 stimulates insulin release in a dose-dependent manner, but this effect is independent of glucose concentrations. Whilst the mechanism of action has yet to be fully established, an increase in beta-cell intracellular calcium is observed with cardiotoxin-1 and action at Kv channels has been hypothesised.^153^ Thus, it could be utilised to gain a better understanding of the important Kv channel-dependent insulin secretory pathway in pancreatic beta cells.^154^ In addition, Moore et al isolated insulin releasing compounds from *Crotalus adamanteus, Crotalus vegrandis and Bitis nasicornis* snakes by gel filtration chromatography. The insulinotropic action appeared to be linked to serine proteinases, phospholipases A_2_ (PLA_2_) and disintegrins within the venom.^155^ PLA_2_ belong to a group of snake toxins with insulinotropic abilities, as exposure results in hydrolysis of membrane phospholipids, the production of arachidonic acid and subsequently insulin secretion.^156,157^ Additionally, disintegrins represent a class of snake peptides that have been implicated in anti-platelet activity but may also possess effects on beta cell insulin secretion.^158^

Finally, a crotamine-like protein was extracted from the venom of the south American rattlesnake *Crotalus durissus cascavella* that displayed glucose-dependent insulin secretory actions.^159^ Crotamine is one of the main toxins of the American rattlesnake and has previously be shown to be non-toxic to human endothelial, fibroblasts and muscle cells.^160,161^ Although the insulinotropic effects have not been fully elucidated, it may be linked to modulation of Na^+^ channels. The putative mechanism of action comes from it inducing membrane depolarisation-dependent muscle contractions by increasing the Na^+^ permeability of skeletal muscle membrane.^162^ The glucose-dependent nature of the peptide could represent a major therapeutic advantage over other classes of antidiabetic drugs, as already noted with exendin-4.

### Wasp and bee venom

**Figure 3.**
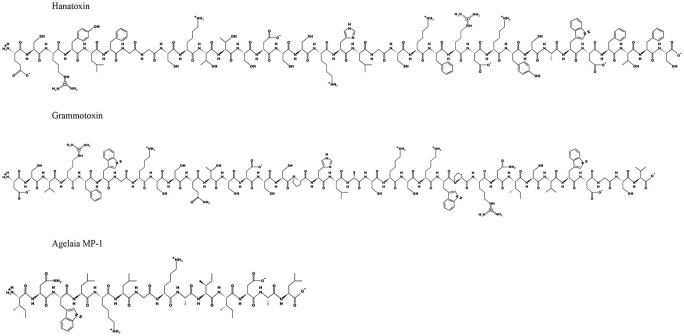
2D structural information for hanatoxin, grammotoxin and agelaia MP-1 based on known amino acid sequences.

The most abundant class of peptides found within wasp venom are known as mastoparans.^163^ It has previously been shown that mastoparans stimulate the release of insulin in the presence or absence of glucose, however phospholipase A_2_ was required for this biological action.^164^ This suggests an interaction between beta cell GPCRs and mastoparan, leading to activation of phospholipase A_2_ and subsequent insulin release.^164^ Furthermore, agelaia MP-1 is a 14 amino acid mastoparan peptide isolated from the venom of the social wasp *Agelaia pallipes pallipes* (Table 3; Figure 3), with 3D structure predicted by computer software packages.^165^ Agelaia MP-1 was shown to induce mast cell degranulation as well as stimulate insulin secretion.^165^ Importantly, insulinotropic actions were not related to beta cell lysis, and interestingly persisted despite inhibition of the Ca^2+^ and K_ATP_ channels, suggesting GPCR interaction and activation of subsequent second messenger cell signalling pathways.^165^ However, there has been some concern about bee venom and its non-specific mode of action and possible adverse side effects.^166^ Nonetheless, melittin is recognised as a potential anti-diabetic agent and studies are underway to reduce or neutralise related toxicity, without detrimentally affecting therapeutic promise. This includes polymer nanoparticle delivery systems appear to significantly reduce, or even annul, toxicity.^167,168^ Further to this, melittin derived from bee venom belongs to a class of gating modifier toxins (GMTS) that modulate voltage-gated ion channels and possibly stimulate insulin secretion through interaction with beta cell K^+^ channels^169^ (Figure 2). A GMTS nanocomplex was formulated to extend duration of action and prevent possible toxicity, with strong electrostatic interactions between negatively charged polyanions, dextran sulphate (DS) and the positively charged melittin.^169^ The formation of this (DS)/melittin nanocomplex successfully increased the half-life of melittin and reduced its acute toxicity in concert with controlling blood-glucose levels for 48 hours in diabetic mouse models.^169^

Moreover, bee venom may not only improve glycaemic control, but can also help wound healing, a classic complication of diabetes.^170^ Bee venom is known for its antimicrobial and anti-inflammatory qualities, suggesting potential for the improvement of this complication.^171^ Therefore, it is not surprising that diabetic rodents treated with bee venom display improved wound healing, in studies that not only improve our understanding of diabetic wound healing processes, but also suggests bee venom as a potential therapeutic option.^171^ The mechanism of anti-inflammatory effects of bee venom are still not fully understood. However, gene expression studies demonstrate ability of bee venom to inhibit lipid accumulation and downregulate adipogenic transcription factors, such as PARγ and C/EBPα.^172^ Furthermore, bee venom was recently shown to improve GLUT-4 expression and insulin sensitivity suggesting bee venom as a potential treatment for chronic inflammation in obesity as well as diabetes.^172^ Indeed, venom from the Egyptian honeybee *Apis mellifera lamarckii*, improved diabetic status in alloxan treated rats.^173^ Beneficial effects were believed to be related to the melittin and phospholipase A_2_ components of the venom.^173^ Finally, diabetes and prolonged periods of hyperglycaemia can lead to protein glycation and impairment of biological activity.^174^ In this respect, honeybee *Apis mellifera* venom reduced the extent of glycation of haemoglobin in an in vitro assay over a 5-week incubation period,^175^ with a mechanism linked to preventing the binding of glucose to haemoglobin, and therefore preventing glycation induced changes in the haemoglobins secondary structure.^175^

### Scorpion venom

As noted above, The Kv channel mode of action on pancreatic beta cells includes repolarisation of the membrane and limiting Ca^2+^ entry into the beta cell.^176,177^ In this regard, there has been identification of beta cell calcium-activated potassium BK (big potassium) channels.^178^ This channel is activated upon Ca^2+^ influx into the beta cell and subsequent membrane depolarisation. In this regard, a peptide extracted from the Eastern Indian red scorpion *Buthus tamulus*, named iberiotoxin is an antagonist of this channel,^137,179^ with its 3D structure available from the uniport number provided (Table 3). Iberiotoxin has been shown to increase the duration of the beta cell action potential by delaying rectifier currents, and subsequently increase insulin secretion in human pancreatic beta cells.^137,179^ However, whilst iberiotoxin has helped with understanding of beta cell secretory dynamics, therapeutic promise could be compromised by lack of specificity, as BK channels also play an important role in the CNS.^180^ Thus, further study into the therapeutic potential and safety profile of iberiotoxin in diabetes is still required.

### Spider venom

Two K^+^ ion channel modifier peptides, named hanatoxin and grammotoxin (Table 3; Figure 3), were successfully extracted from the venom of the Chilean rose tarantula *Grammostola rosea*.^137,181^ Hanatoxin is a 35 amino acid peptide with 6 cysteine residues^181^ and is described as a potent inhibitor of the Kv2.1 component of the K^+^ repolarisation beta cell channel^181,182^ (Figures 2 and 3). Interestingly, the peptide is known to insert itself into the phospholipid membrane hydrocarbon core, without the requirement of pore formation, before it then encounters and inhibits the Kv channel inside the membrane phospholipid.^183,184^ It has been demonstrated that the peptide can also inhibit the Kv2.2 channels, but with a lower infinity.^137^ In keeping with this, hanatoxin increases glucose-stimulated insulin secretion and calcium oscillators in both mouse and human islets.^2^ Grammotoxin exhibits 43% amino acid sequence homology to hanatoxin and is believed to have a similar mechanism (Figure 3), but it binds to the same Kv channels with lower affinity^185,186^ (Figure 2). However, whilst comparison of the 3D structures of hanatoxin and grammotoxin does reveal some clear conservation of structures between both peptides, slight differences in the surface shape and distribution of the charged residues may help explain the lower binding affinity of grammotoxin.^186^ Following on from this, guangtoxin (GxTX) has been extracted from the *Plesiophrictus guangxiensis* tarantula^187,188^ (Table 3). GxTX has been shown to broaden the length of the glucose-induced beta cell action potential and increase related Ca^2+^ oscillations through interaction with the Kv channel, whilst being ineffective at low glucose levels, thus limiting potential to cause hypoglycaemia.^187,188^

Although exciting in terms of potential therapeutic value, a disadvantage is the widespread expression of Kv channels, and it may prove challenging to specifically target beta cell Kv channels.^137^ However, inhibition of the Kv2.1 channel has been demonstrated to promote beta cell survival in streptozotocin-diabetic mice, demonstrating clear potential as a type 2 diabetes therapeutic.^189^ Furthermore, in comparison to snake venoms, the study and application of spider venom peptides as potential diabetes therapeutics is somewhat limited. This is most likely a direct reflection of the amount of venom that can be milked, and subsequently analysed and tested, from a snake as opposed to a spider. However, it should be acknowledged that the majority of current peptide discovery efforts focus more on genomics and transcriptomics to identify potentially interesting sequences, which are then synthesised or expressed, rather than relying on extraction from the animal. Nonetheless, spider venoms are conservatively predicted to contain more than 10 million bioactive peptides, many of which have never been investigated, making them a valuable resource for peptide-based drug discovery.^190^ Additionally, although venom-derived peptides are generally extremely enzymatically stable in the circulation, their administration will likely still require parenteral delivery to avoid proteases activity within the stomach. However, major strides have been taken in terms of oral formulation of peptides, including clinical trials with insulin, GLP-1, calcitonin, parathyroid hormone and vasopressin.^191^ Given the wealth of peptides present in spider venom and this prospect of oral delivery, they provide an ideal subject matter for exploration in relation to insulin secretion from pancreatic beta cells, and merit much further study in this regard.

## Conclusion

Advances in biological methodologies has allowed for the discovery and characterisation of more animal venom-derived compounds with potential therapeutic or cosmeceutical application. The success of clinically approved drugs in this field, as well as others undergoing clinical trials and some well-established cosmeceuticals such as Botox, only serves to strengthen this viewpoint. In particular, approval of exendin-4 as a first in class therapeutic for diabetes, highlights the possibility of future antidiabetic agents being derived directly from venom constituents. Although this is an extremely exciting area of drug discovery, further studies are needed to dissect the underlying mechanisms, safety profile and possible requirement for tissue-targeting of currently characterised compounds in this field. Indeed, off-target effects may see many of these toxin products utilised only as research tools to better understand pancreatic beta-cell function, rather than clinically approved therapeutics or useful cosmeceuticals. However, in particular, more investigation into the unquestionable untapped therapeutic potential of spider venom peptides is still required.
